# *Cardiocondyla pirata* sp. n. – a new Philippine ant with enigmatic pigmentation pattern (Hymenoptera, Formicidae)

**DOI:** 10.3897/zookeys.301.4913

**Published:** 2013-05-17

**Authors:** Bernhard Seifert, Sabine Frohschammer

**Affiliations:** 1 Senckenberg Museum für Naturkunde Görlitz, PSF 300154, D-02806 Görlitz; 2 Biologie I, Universität Regensburg, Universitätsstraße 31, D-93053 Regensburg

**Keywords:** *Cardiocondyla*, ergatoid males, Indo-Malayan region

## Abstract

A new species of the ant genus *Cardiocondyla* Emery, 1869 – *Cardiocondyla pirata* **sp. n.** – is described from the Philippines. The species belongs to an Indo-Malayan group of six species that is characterized by workers having a strongly bilobate postpetiolar sternite and a thickset mesosoma with strongly convex dorsal profile as well as wingless, ergatoid males with sickle-shaped mandibles. The female castes show a pigmentation pattern not known from any ant worldwide. If having any adaptive value, a possible function of this structure is supposed to be visual dissolution of body shape in order to irritate predators.

## Introduction

105 available names are listed in the ant genus *Cardiocondyla* Emery, 1869 and 68 of these are currently considered to designate bona species (Bolton 2012). While there is a rather good taxonomic knowledge of the species groups distributed in the Palaearctic (Seifert 2003), the situation in the Oriental, Indo-Malayan and Australasian faunal regions is poorly known. This is indicated by the fact that there is a minimum of 15 morphologically well-separated, but yet undescribed species from this region in the collection of the Senckenberg Museum of Natural History Goerlitz (unpublished protocols of the senior author).

During a field study of ants in the Hortarium of the Los Baños University / Philippines, one of the authors collected two nest samples of a *Cardiocondyla* species that shows a pigmentation pattern unknown in any ant worldwide. The new species belongs to a species group that is distributed from Thailand across the whole Indo-Malayan region and contains a minimum of six yet undescribed species. This species group is characterized by a strongly bilobate postpetiolar sternite, a thickset mesosoma with strongly convex dorsal profile and wingless, ergatoid males with sickle-shaped mandibles.

## Methods

### Recording of morphological data

Nineteen morphometric characters were investigated. In bilaterally recorded characters, arithmetic means of both body sides were calculated. All measurements were made on mounted and dried specimens using a pin-holding stage, permitting full rotations around X, Y, and Z axes. A Leica M165C high-performance stereomicroscope equipped with a 2.0 planapochromatic objective (resolution 1050 lines/mm) was used at magnifications of 120–384×. The mean relative measuring error over all magnifications was 0.3%. A Schott KL 1500 cold–light source equipped with two flexible, focally mounted light–cables, providing 30°–inclined light from variable directions, allowed sufficient illumination over the full magnification range and a clear visualization of silhouette lines. A Schott KL 2500 LCD cold–light source in combination with a Leica coaxial polarized–light illuminator provided optimum resolution of tiny structures and microsculpture at highest magnifications. Simultaneous or alternative use of the cold-light sources depending upon the required illumination regime was quickly provided by regulating voltage up and down. A Leica cross-scaled ocular micrometer with 120 graduation marks ranging over 52 % of the visual field was used. To avoid the parallax error, its measuring line was constantly kept vertical within the visual field (Seifert 2002). Measurements of body parts always refer to real cuticular surface and not to the diffuse pubescence surface.

Z-stack photographs were made with a Leica Z6 APO photomicroscope equipped with 2.0× planapochromatic objective and the automontage software Leica application suite version 3.

### Definition of numeric characters

More detailed explanations of the character recording are given in Seifert (2003). Here we repeat the verbal definitions:

**CL** maximum cephalic length in median line; the head must be carefully tilted to the position yielding the true maximum; excavations of hind vertex and/or clypeus reduce CL.

**CW** maximum cephalic width; in *Cardiocondyla*, the maximum is found usually across and including the eyes, exceptionally posterior of the eyes.

**CS** cephalic size; the arithmetic mean of CL and CW, used as a less variable indicator of body size.

**dFOV** mean inner diameter of foveolae or mesh-like surface structures on vertex at about half way between the median line of head and the inner eye margin. These structures are either real foveolae or meshes of a reticulum and usually have the base of a decumbent pubescence hair in their centre. In species whose foveolae or mesh-like structures are reduced (e.g. in the *Cardiocondyla stambuloffii* group) the mean diameter of the small punctures or tubercles at hair bases is measured as dFOV. At least seven measurements are averaged.

**EYE** eye-size: the arithmetic mean of the large (EL) and small diameter (EW).

**FRS** distance of the frontal carinae immediately caudal of the posterior intersection points between frontal carinae and the lamellae dorsal of the torulus. If these dorsal lamellae do not laterally surpass the frontal carinae, the deepest point of scape corner pits may be taken as reference line. These pits take up the inner corner of scape base when the scape is fully switched caudad and produce a dark triangular shadow in the lateral frontal lobes immediately posterior of the dorsal lamellae of scape joint capsule.

**MpG** Depth of metanotal groove or depression, measured from the tangent connecting the dorsalmost points of promesonotum and propodeum.

**ML** mesosoma length in the alates; measured in lateral view from the caudalmost portion of propodeum to the frontalmost point of the anterior pronotal slope (i.e., not to the frontalmost point of the whole pronotum that is usually concealed by the occiput!).

**MW** maximum mesosoma width of alates frontal of the tegulae.

**PEH** maximum petiole height. The straight section of ventral petiolar profile at node level is the reference line perpendicular to which the maximum height of petiole node is measured at node level.

**PEL** diagonal maximum length of petiole in lateral view, measured from anterior corner of subpetiolar process to dorsocaudal corner of caudal cylinder.

**PEW** maximum width of petiole.

**PLG** mean length of pubescence hairs on dorsum of first gaster tergite as arithmetic mean of 6 measurements at least.

**PPH** maximum postpetiole height; the lateral suture of dorsal and ventral sclerites is the reference line perpendicular to which the maximum height of postpetiole is measured.

**PPW** maximum width of postpetiole.

**PoOc** postocular distance. Use a cross-scaled ocular micrometer and adjust the head to the measuring position of CL. Caudal measuring point: median occipital margin; frontal measuring point: median head at level of posterior eye margin. Note that many heads are asymmetric; therefore average the left and right postocular distance.

**SL** maximum straight line length of scape excluding the articular condyle given as the arithmetic mean of both scapes.

**SPBA** the smallest distance of the lateral margins of the spines at their base. This should be measured in dorsofrontal view, since the wider parts of the ventral propodeum do not interfere the measurement in this position. If the lateral margins of spines diverge continuously from the tip to the base, a smallest distance at base is not defined. In this case, SPBA is measured at the level of the bottom of the interspinal meniscus.

**SP** maximum length of propodeal spines; measured in dorsofrontal view along the long axis of the spine, from spine tip to a line, orthogonal to the long axis, that touches the bottom of the interspinal meniscus. Left and right SP are averaged. This mode of measuring is less ambiguous than other methods but yields higher spine length values in species with reduced spines.

**sqPDG** square root of pubescence distance on dorsum of first gaster tergite. The number of pubescence hairs n crossing a transverse measuring line of length L is counted; hairs just touching the line are counted as 0.5. The pubescence distance PDG is then given by L/n. In order to normalize the positively skewed distributions, the square root of PDG is calculated. Exact counts are promoted by clean surfaces and flat, reflection-reduced illumination directed slightly skew to the axis of the pubescence hairs. Counting is performed at a magnification of 384×. Tergite pubescence is easily torn-off in *Cardiocondyla*. An effort should be made to evaluate undamaged surface spots. In specimens with mostly removed pubescence, PDG can be calculated from the mean distance of hair base pits (BD) and PLG using the formula PDG = BD^2^ /PLG.

## Results

### Cardiocondyla pirata sp. n.

urn:lsid:zoobank.org:act:C1BA401A-3510-494E-B66F-CC5FCCF030A1

http://species-id.net/wiki/Cardiocondyla_pirata

#### Etymology.

The species epithet refers to the black ribbon across the eye reminiscent of a pirate’s blindfold.

#### Type material.

Holotype worker labeled “PHI: 14.1643°N, 121.2375°E, Los Banos, University Park, 58 m, Hortarium, Frohschammer 2008.07.23 #39” and ”Holotype *Cardiocondyla pirata* Seifert, 2013”; 4 workers, 3 dealate gynes and 1 ergatoid male labeled “PHI: 14.1643°N, 121.2375°E, Los Banos, University Park, 58 m, Hortarium, Frohschammer 2008.07.23 #39” and ”Paratype *Cardiocondyla pirata* Seifert, 2013”; 3 workers labeled “PHI: 14.1643°N, 121.2375°E, Los Banos, University Park, 58 m, in hole of a stone at riverside, Frohschammer 2008.07.23 #32” and ”Paratype *Cardiocondyla pirata* Seifert, 2013”; all material in Senckenberg Museum of Natural History Goerlitz.

**Description and differential diagnosis.** Measurements and indices in square brackets are arithmetic mean (see also Table 1).

**Figure 1.**
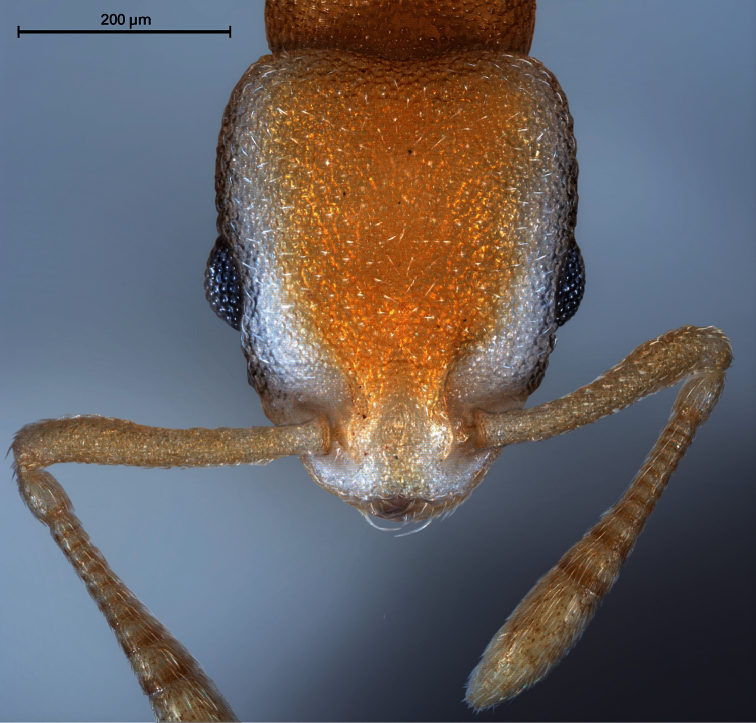
Head of holotype worker in dorsal view.

**Figure 2.**
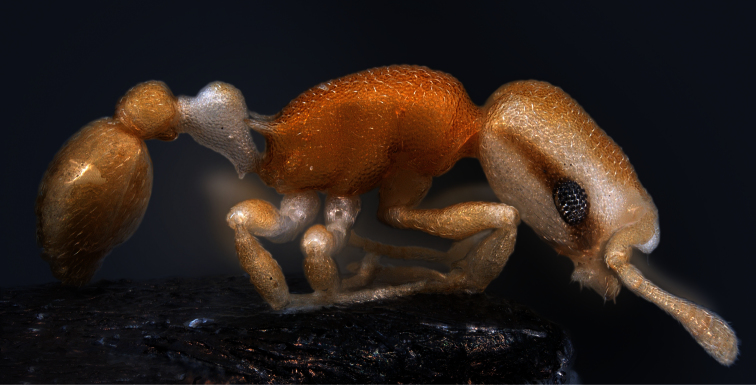
Lateral aspect of holotype worker.

**Worker** (Figs 1–2, Table 1): Unmistakable pigmentation pattern for an ant worldwide. Lateral head at horizontal level of eye with an extended, longitudinal, dark brown ribbon that is as broad as the eye; this ribbon is flanked below and above by broad bands without any pigment (as result appearing whitish). Vertex, scape, postpetiole, gaster, procoxae, tibiae and femora except their proximal and distal portions light yellowish brown. Mesosoma light orange brown. Petiole, meso- and metacoxae, clypeus, spines, funiculus as well as proximal and distal portions of femora without pigmentation (appearing whitish). Very small size [CS 397 µm]. Head moderately elongated [CL/CW 1.132]. Postocular distance relatively small [PoOc/CL 0.408]. Eye rather small [EYE/CS 0.226]. With maximum CL and CW in visual plane, outlines of head roughly heart-shaped, with strongly concave posterior margin and an almost straight anterior clypeal margin (a distinct concavity appears after a tilt to frontodorsal viewing position when the three clypeal macrosetae become fully visible). Frontal carinae much more closely spaced than in any related species [FRS/CS 0.242], subparallel and slightly diverging frontal of the FRS level. Mesosoma thickset, its dorsal profile evenly convex. Anterior pronotum in dorsal view rounded, without pronounced corners. Propodeal spines straight and much shorter than in any related species [SP/CS 0.208], in dorsal view slightly diverging, in lateral view straight and with their axis deviating by 40° from longitudinal axis of mesosoma. Petiole in lateral view rather massive, clearly higher than postpetiole, with a short peduncle, a slightly concave anterior profile and a convex dorsal node that steeply slopes down to the caudal cylinder; the node in dorsal view semiglobular and slightly wider than long. Postpetiole in dorsal view with a straight or slightly concave anterior margin, rounded sides and much wider than long; its sternite with pronounced anterolateral corners that are formed by bilateral lobes which strongly protrude compared to anteromedian level. Whole surface of head, mesosoma and petiole with a very fine (mesh diameter on vertex only 8-9 µm) but deeply sculptured reticulum, thus appearing at lower magnifications perfectly matt. Postpetiole less deeply sculptured. Scapes, coxae, femora and tibiae with fine microreticlum and appearing matt at lower magnifications. First gaster tergite very finely microreticulate-shagreened, also appearing matt at lower magnifications. All cuticular surfaces including those of the appendages with decumbent, dilute pubescence. Pubescence on 1st gaster tergite much longer and denser than in any related species [PLG/CS 7.21%, sqPDG 3.92], on anterior surface directed caudad and on posterior one caudomediad. For morphometric data of 6 workers (three from each sample) see Table 1.

**Table 1.** Morphometric data of workers, gynes and a male of *Cardiocondyla pirata* sp. n. Worker data of five undescribed, most closely related species are given - the strings in capitals are code designations of these species in the files of B. Seifert. Arrangement of data: arithmetic mean ± standard deviation [lower extreme, upper extreme]. Measurements of *Cardiocondyla pirata* workers radically differing from those of related species are given in bold.<br/>

	**sp.: ARGE, ARGY, ARPI, LATI, MISE**	***Cardiocondyla pirata* sp. n.**		
	**worker (n=174)**	**worker (n=6)**	**gyne (n=3)**	**male (n=1)**
CS [µm]	423 ± 45<br/> [358,556]	397 ± 4<br/> [392,402]	437 ± 3<br/> [433,440]	341
CL/CW	1.113 ± 0.027<br/> [1.040,1.182]	1.132 ± 0.010<br/> [1.120,1.147]	1.152 ± 0.005<br/> [1.146,1.155]	1.070
SL/CS	0.829 ± 0.018<br/> [0.785,0.892]	0.807 ± 0.005<br/> [0.800,0.816]	0.792 ± 0.011<br/> [0.782,0.804]	0.707
PoOc/CL	0.418 ± 0.011<br/> [0.389,0.455]	0.408 ± 0.005<br/> [0.404,0.416]	0.401 ± 0.002<br/> [0.398,0.402]	0.420
EYE/CS	0.228 ± 0.009<br/> [0.204,0.247]	0.226 ± 0.002<br/> [0.223,0.229]	n.r.	0.210
dFov [µm]	16.4 ± 1.8<br/> [10.9,20.0]	8.6 ± 0.3<br/> [8.2,9.0]	13.7 ± 2.8<br/> [10.9,16.5]	n.r.
FRS/CS	0.325 ± 0.020<br/> [0.263,0.357]	**0.242** ± 0.007<br/> [0.235,0.254]	0.248 ± 0.006<br/> [0.242,0.254]	0.347
MW/CS	n.r.	n.r.	0.797 ± 0.016<br/> [0.784,0.815]	0.789
ML/CS	n.r.	n.r.	1.301 ± 0.005<br/> [1.297,1.306]	1.161
MGr/CS	0.38 ± 0.36<br/> [0.0,1.48]	0.0 ± 0.0<br/> [0,0]	n.r.	0.0<br/>
SPBA/CS	0.358 ± 0.020<br/> [0.303,0.414]	0.373 ± 0.005<br/> [0.364,0.378]	0.438 ± 0.015<br/> [0.422,0.452]	0.351
SP/CS	0.366 ± 0.062<br/> [0.232,0.476]	**0.208** ± 0.004<br/> [0.204,0.214]	0.229 ± 0.005<br/> [0.223,0.232]	0.161
PEW/CS	0.310 ± 0.020<br/> [0.267,0.362]	0.359 ± 0.004<br/> [0.355,0.367]	0.392 ± 0.002<br/> [0.391,0.394]	0.419
PPW/CS	0.459 ± 0.028<br/> [0.397,0.531]	0.468 ± 0.004<br/> [0.462,0.475]	0.532 ± 0.007<br/> [0.524,0.537]	0.582
PEH/CS	0.355 ± 0.018<br/> [0.315,0.422]	0.343 ± 0.006<br/> [0.335,0.354]	0.391 ± 0.006<br/> [0.386,0.398]	0.387
PPH/CS	0.299 ± 0.031<br/> [0.231,0.359]	0.320 ± 0.008<br/> [0.308,0.328]	0.361 ± 0.005<br/> [0.358,0.366]	0.372
sqPDG	5.34 ± 1.04<br/> [3.42,8.25]	3.92 ± 0.13<br/> [3.74,4.12]	2.63 ± 0.07<br/> [2.55,2.68]	3.66
PLG/CS [%]	4.37 ± 1.21<br/> [2.14,6.75]	**7.21**± 0.26<br/> [6.87,7.54]	7.68 ± 0.24<br/> [7.49,7.95]	9.53

**Figure 3.**
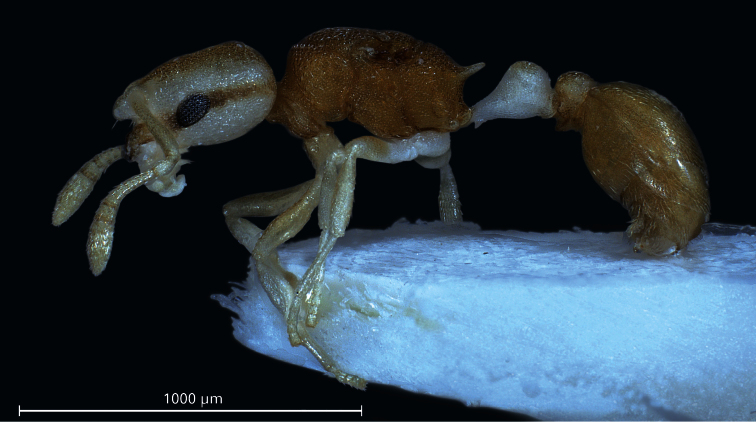
Lateral aspect of a paratype gyne. Postpetiole and gaster are distorted to a ventrolateral viewing position making visible both lobes of postpetiolar sternite.

**Figure 4.**
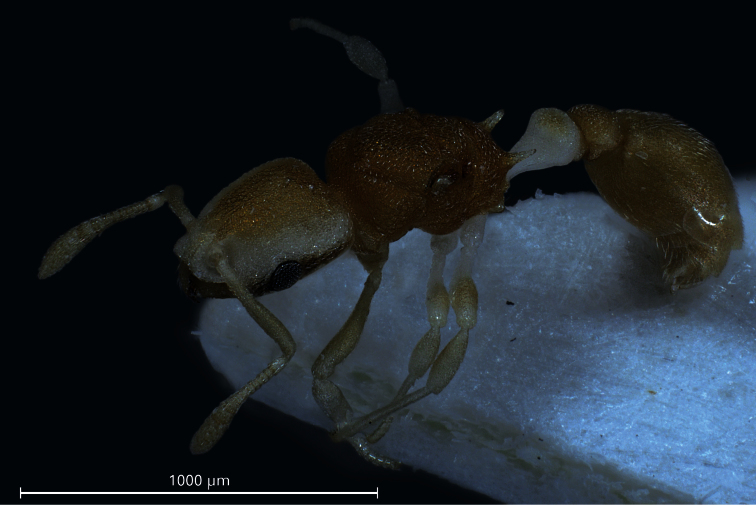
Dorsolateral aspect of a paratype gyne. Only one lobe of postpetiolar sternite is visible.

**Gyne** (Figs 3, 4, Table 1): Unmistakable pigmentation pattern most similar to that described in the worker. Very small size [CS 437 µm]. Head shape comparable to worker but head more elongated, CL/CW 1.152. Postocular distance relatively small [PoOc/CL 0.401]. Frontal carinae much more closely spaced than in any related species [FRS/CS 0.248], subparallel and slightly diverging frontal of the FRS level. Mesosoma shorter than in the next related species [ML/CS 1.301]. Propodeal spines straight and much shorter than in any related species [SP/CS 0.229], in dorsal view slightly diverging, in lateral view straight and with their axis deviating by 30° from longitudinal axis of mesosoma. Petiolar and postpetiolar shape comparable to worker but with the usual gyne-specific shape transformation: increased segment width and height relative to their length and postpetiole in dorsal view with a more concave anterior margin. All body surfaces appearing matt at lower magnification. Cuticular sculpture on all body surfaces similar to worker but several larger foveolae of 15–21 µm diameter, showing a central tubercle as basis of a pubescence hair, are interspersed within the fine reticulum of head and mesosoma. All cuticular surfaces, including those of the appendages, with decumbent, dilute pubescence. Pubescence on 1st gaster tergite much longer and denser than in any related species (PLG/CS 7.68%, sqPDG 2.63), on anterior surface directed caudad and on posterior one caudomediad. For morphometric data of 3 gynes see Table 1.

**Figure 5.**
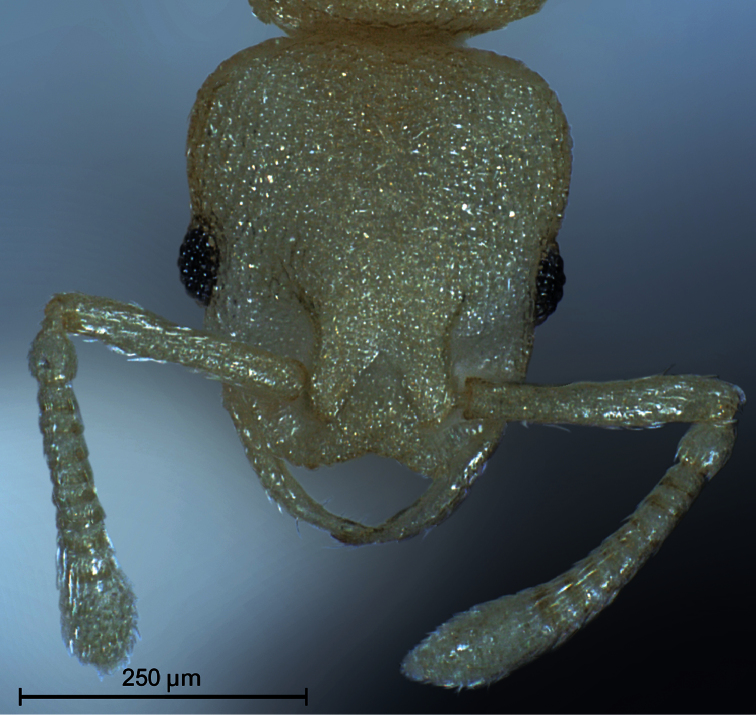
Head of the paratype male in dorsal aspect.

**Figure 6.**
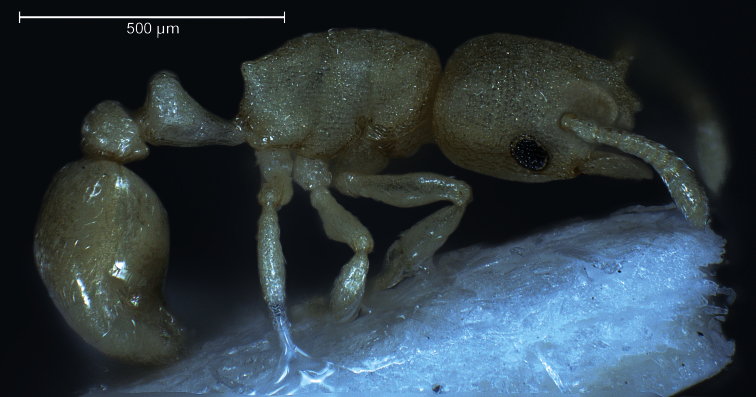
Lateral aspect of the paratype male. The head is distorted to a dorsolateral viewing position.

**Male** (Figs 5, 6, Table 1): Ergatoid. With exception of the blackish eyes, whole body concolorous pale yellowish. Nanitic size [CS 341 µm]. Antennae with 11 segments. Mandibles long and sickle-shaped, toothless. Head short [CL/CW 1.070]. Postocular distance relatively small [PoOc/CL 0.420]. Eye small [EYE/CS 0.210]. With maximum CL and CW in visual plane, outlines of head roughly trapezoid, with only weakly concave posterior margin and sides of head converging frontad. Anterior clypeal margin with a broad angular excision forming an angle of about 145°. Clypeus strongly extending caudad to about half length of frontal carinae. Frontal carinae much more distant than in female castes [FRS/CS 0.347] and almost parallel. Mesosoma very thickset and short, its dorsal profile evenly convex. With mesosoma in dorsal view, anterior pronotum rounded, without pronounced corners; pronotum and anterior mesonotum nearly twice as wide than the distance between the parallel sides of dorsal propodeum. Propodeal spines short, reduced to triangular dents. Petiole in lateral view more elongated, with a distinct peduncle, a concave anterior face and a high and short node that shows a rounded dorsum and falls almost perpendicularly down to the caudal cylinder. Petiolar node in dorsal view nearly 2.5fold wider than long, in anterior view strongly diverging dorsad and with an emarginate dorsal crest. Postpetiole in dorsal view 1.7 fold wider than its median length, with a slightly concave anterior margin and rounded sides; its sternite with pronounced anterolateral corners that are formed by bilateral lobes which strongly protrude compared to anteromedian level. Sculpture on all body surfaces similar to worker but sculpture and microsculpture on postpetiole and 1^st^ gaster tergite less developed - as result these surfaces moderately shining. All cuticular surfaces including those of the appendages with decumbent, dilute pubescence. Pubescence on 1st gaster tergite much longer than in worker [PLG/CS 9.53%, sqPDG 3.66], on anterior surface directed caudad and on posterior one caudomediad. For morphometric data of the single investigated male see Table 1.

#### Comments.

*Cardiocondyla pirata* sp. n. cannot be confused with any ant worldwide because of its unique pigmentation pattern. This clear identification is supported by diagnostic structural and shape characters: there is no overlap with the five other undescribed species of this species group in the characters FRS/CS, SP/CS and PLG/CS and there is little overlap in PEW/CS and sqPDG (Table 1).

One complete colony consisting of three dealate queens, 15 workers and brood was collected in the field. From a second colony, only a sample was taken in EtOH. The first colony produced over 20 female sexuals and one ergatoid male in the lab, but thereafter died. Hence, there are no long-term observations on the life history of this interesting species. Considering the situation in related species of the Oriental and Indo-Malayan region (Heinze et al. 2010, Oettler et al. 2010), we may predict for *Cardiocondyla pirata* sp. n. the following biological traits: (a) there are only ergatoid males - winged males, which are an ancestral trait in *Cardiocondyla*, are no longer developed, (b) ergatoid males are long-lived, mate always inside the nest and try to kill rivals using their sickle-shaped mandibles in order to monopolize the matings and (c) nests should contain 1–4 queens.

The adaptive significance of the extraordinary pigmentation pattern of these tiny ants remains a puzzle. The poor resolution of the visual system (workers and males have only 50–65 ommatidiae per eye) and the dominance of chemical and tactile recognition cues in these ants as well as the fact that mating happens in darkness of the nest certainly exclude a function as an intraspecific recognition signal. A possible function could be visual dissolution of body shape by alternating dark and light pigment in order to escape the attention of a predator. The unpigmented petiole in particular, being in living condition rather translucent, permits the visual impression that anterior and posterior body are separate objects. Remains the question: Which predator with a high-performance visual system could consume these tiny ants?

## Supplementary Material

XML Treatment for Cardiocondyla pirata
